# Th9 and IL9 in Chronic Superior Airway Inflammation: A Narrative Review

**DOI:** 10.3390/biomedicines14051026

**Published:** 2026-04-30

**Authors:** Mihai Dumitru, Ovidiu Berghi, Gabriela Musat, Crenguta Serboiu, Alina Oancea, Alina Gabriela Berghi, Adina Zamfir-Chiru-Anton, Daniela Vrinceanu

**Affiliations:** 1 ENT Department, Bucharest University Emergency Hospital, Carol Davila University of Medicine and Pharmacy, 050474 Bucharest, Romania; orldumitrumihai@yahoo.com (M.D.); vrinceanudana@yahoo.com (D.V.); 2 Allergology and Immunology Department, Saint Mary Laboratories and Clinics, 011013 Bucharest, Romania; oberghi@yahoo.com; 3 ENT Department, Saint Mary Clinical Hospital, Carol Davila University of Medicine and Pharmacy, 050474 Bucharest, Romania; 4 Molecular Biology and Histology Department, Carol Davila University of Medicine and Pharmacy, 050474 Bucharest, Romania; crengutas@yahoo.com; 5 Elias Clinical Hospital, Carol Davila University of Medicine and Pharmacy, 050474 Bucharest, Romania; dr.alina.oancea@gmail.com (A.O.); radualinag@yahoo.com (A.G.B.); 6 ENT Department, Grigore Alexandrescu Clinical Hospital, 011743 Bucharest, Romania

**Keywords:** Th9, IL9, allergic rhinitis, rhinosinusitis

## Abstract

Inflammation at the superior airway level has multiple manifestations, and allergic rhinitis and chronic rhinosinusitis with or without polyps are two of the most frequent and troublesome of them, with innate and adaptive immunity being implicated. Dendritic cells, epithelial cells, neutrophils, macrophages, mucosal mast cells, eosinophils, basophils, innate lymphoid cells (ILCs), and NK cells are the players in innate immunity, while regulatory T (Treg), TH1, TH2, TH17, T follicular helper, and B cells are components of the adaptative immune system. Th9 cells, a subset of T helper cells discovered in 2008 that produce interleukin-9 (IL-9), play a vital role in the adaptive immune response and have advantageous and harmful effects in different diseases due to the induction pattern. We queried international databases for current, up-to-date information regarding the interplay between interleukin 9 (IL-9) and helper T cells (especially Th9 cells), and by other immune cells. Interleukin-9 has multiple immunological functions, acting on various target cells through its specific receptor (IL-9R), such as the following: the regulation of allergic (Th2-type) immune responses; effects on epithelial and mucosal cells, mast cells, and eosinophils; chronic inflammation; and autoimmunity. Thus, there is a further need to translate laboratory findings into clinical practice regarding IL-9.

## 1. Introduction

**Figure 1:**
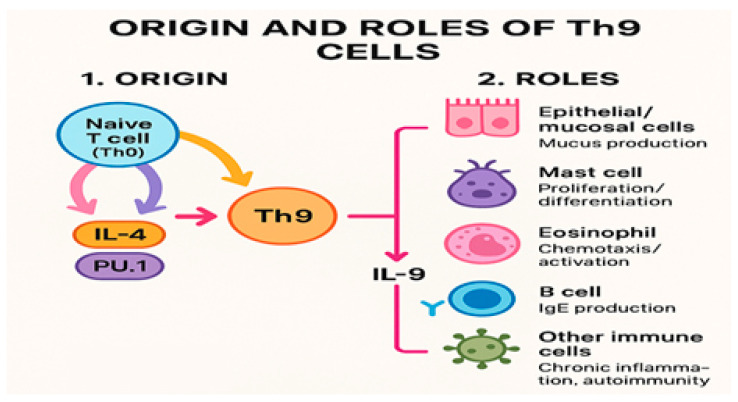
Summary of Th9 origin and IL-9-mediated roles. Th0 cells differentiate into Th9 cells via IL-4 and PU.1. Th9-derived IL-9 drives mucus production (epithelial/mucosal cells), mast cell proliferation/differentiation, eosinophil chemotaxis/activation, and B-cell IgE production and contributes to chronic inflammation/autoimmunity. Th0—naïve CD4^+^ T helper cell; Th9—T helper 9 cell; IL-4—interleukin-4; PU.1—transcription factor PU.1; IL-9—interleukin-9; IgE—immunoglobulin E.

Th9 cells are a subset of CD4+ Th cells that were first identified in 2008 by the teams of Veldhoen and Dardalhon. These cells develop from naïve T cells and secrete interleukin (IL)-9 [1,2]. The classification of Th9 cells as a distinct lineage remains debated, as no unique lineage-determining transcription factor has been identified, and some researchers propose that Th9 cells found in vivo represent a transient subset of Th2 cells, rather than a distinct lineage [2,3]. Unlike Th2 cells, Th9 cells do not strongly express IL-4, IL-5, or IL-13, which is a key distinguishing feature (Figure 1).

The development of Th9 cells relies on critical transcription factors, such as interferon-regulatory factor 4 (IRF4) and PU.1. The process begins with the differentiation of naïve Th0 cells into Th2 cells, characterized by upregulated IRF4 expression and suppressed PU.1 activity. The subsequent activation of the Smad3/Smad4 and IRF4 pathways enables the transformation of Th2 cells into Th9 cells under the influence of transforming growth factor beta (TGFβ), which alters the cytokine secretion profile from an IL-4-dominant phenotype to an IL-9-dominant one [1,2].

Th9 cells are implicated in various diseases, including allergic and pulmonary disorders such as asthma, chronic obstructive airway disease (COPD), chronic rhinosinusitis, nasal polyps, and pulmonary hypoplasia, and they primarily produce IL-9, a cytokine critical for regulating autoimmune and allergic responses, in response to activation by IL-4 and TGFβ. In addition to IL-9, Th9 cells may secrete other cytokines, including IL-13, IL-21, and IL-10. Circulating Th9 cells correlate with plasma IgE levels and influence eosinophils, mediating cytotoxic and inflammatory responses during allergic inflammation [3].

IL-9, discovered in the late 1980s, is a 144-amino acid glycoprotein that signals through a receptor complex containing the common γ chain and is a hallmark of Th9 cells [3,4]. In mice, the *Il9* gene is located on chromosome 5 within the TH2 cytokine cluster (IL-3, IL-4, IL-5, GM-CSF, and IL-13) at the 5q31–35 region, while the *Il9r* gene resides on the sex chromosomes (Xq28 and Yq1217). IL-9 is mainly produced by CD4+ Th cells, type-2 innate lymphoid cells (ILC2s), and mast cells, with smaller contributions from regulatory T cells (Tregs), Th17 cells, Th2 cells, B cells, and neutrophils.

The IL-9 receptor (IL-9R) is expressed on various immune cells, including T cells, ILC2s, B cells, and myeloid cells, and is a heterodimeric receptor composed of IL-9Rα and the common γ chain (γC), with the latter shared by the IL-4, IL-7, IL-2, and IL-15 receptors. The IL-9Rα chain, however, is specific to IL-9 and exists in soluble and membrane-bound forms.

IL-9 functions as a growth factor for T cells, enhances IgE production by B cells, induces mucus production in epithelial cells, and promotes mast cell proliferation and differentiation. Elevated IL-9 levels are frequently observed in the lungs of individuals with airway inflammation and hyperresponsiveness, characteristic of type-2-high asthma, and this asthma phenotype is associated with eosinophilia, mast cell infiltration, and increased mucus production [1,2,3].

During allergic inflammation, IL-9 stimulates IgE secretion by B cells, recruits and activates mast cells, promotes eosinophil chemotaxis, and induces mucin production in lung epithelial cells. While multiple T helper subsets produce IL-9, including Th9, Th17, and Th2 cells, the role of each subset in inflammation and immunity varies. The contribution of Tregs to IL-9 production remains under investigation. Th2-derived IL-9 has been linked to asthma pathogenesis and demonstrates pro- and anti-inflammatory effects, similar to IL-27, and its activity can be modulated by tissue microenvironments and cytokine interactions [3]. In CD4 T cells, IL-9 promotes mTOR activation, aerobic glycolysis, and proliferation, and it reinforces its own expression [4].

Human and mouse Tfh cells express the IL-9 receptor, and signaling through this receptor enhances Bcl6 expression in Tfh cells, thereby maintaining their functional integrity. Given the limited IL-9 receptor signaling capacity in Tfr cells, IL-9 may promote germinal center (GC) formation by modulating the functional balance between Tfh and Tfr cells. Notably, a subset of Tfh cells can produce IL-9 themselves, indicating a potential autocrine mechanism within this population. Additionally, memory B cells are capable of producing IL-9, which, in turn, supports Tfh cell maturation and GC formation [5]. IL-9 deficiency significantly attenuated HDM-induced airway hyperresponsiveness and reduced inflammatory cell infiltration in the lung parenchyma and airways, and the absence of IL-9 also led to decreased eosinophil recruitment and goblet cell hyperplasia following HDM exposure. Furthermore, IL-9 deficiency diminished cytokine production in lung homogenates. In HDM-challenged mice, the loss of IL-9 limited the accumulation and proliferation of ILC2s, Th2 cells, and mast cells within lung tissue, highlighting the pivotal role of IL-9 in orchestrating type-2 immune responses during allergic airway inflammation [6]. Short-term exposure to IL-9 or fetal bovine serum negatively impacted the MRGPRX2 expression from consenting healthy adults in accordance with protocols approved by the local institutional review board at UZ/KU Leuven by Leven et al. [7]. IL-9R is consistently expressed throughout various stages of mast cell (MC) development. IL-9 drives tissue-specific MC expansion and directly acts on bone marrow MCs to enhance their proliferative capacity, and it also promotes the CCR2-dependent recruitment of bone marrow MCs to allergic lung tissue. Furthermore, T cell-derived IL-9 stimulates MC expansion in the allergic lung, and MC-mediated airway hyperresponsiveness relies on this T cell-derived IL-9 signaling [8].

The IL-9 locus is linked to Th2 cytokine loci (IL-4, IL-5, IL-13) and shows increased chromatin acetylation and reduced H3K27 trimethylation in Th9 cells, indicating active transcription. Three conserved noncoding sequences (CNSs) regulate this region, with CNS1 serving as the main transcription factor binding site. When naïve CD4^+^ T cells are activated by IL-2, IL-4, and TGF-β, they produce IL-9, mainly driven by TGF-β and IL-2. TGF-β induces PU.1, which promotes Th9 differentiation and suppresses Th2 cell development. PU.1, together with Smad proteins, enhances IL-9 gene activation while inhibiting Th2 cytokines by limiting IRF-4 and GATA3 activity. As a result, Th9 cells display high IL-9 expression and reduced Th2 cytokine production [9]. TL1A, an epithelial cytokine expressed in the alveolar epithelia and airway basal cells of healthy and asthmatic lungs, is also present in mouse alveolar epithelia under steady-state conditions. TL1A acts synergistically with IL-33 to drive the emergence of an IL-9-producing ILC9 phenotype from lung ILC2s. This ILC9 state represents a transient IL-9^high GATA3^low, multicytokine-producing phase of activated ILC2s. In vivo, TL1A and IL-33 cooperate to induce IL-9^high ILC2s, with endogenous IL-33 essential for their development during the early stages of allergic airway inflammation, while endogenous TL1A serves as an epithelial alarmin, promoting the early induction of IL-9^high ILC2s, following allergen exposure. These ILC9 cells possess an enhanced ability to initiate IL-5-dependent allergic airway inflammation [10].

This narrative review synthesizes the current evidence on the role of the Th9–IL-9 axis in allergic rhinitis and chronic rhinosinusitis and evaluates its potential as a biomarker or therapeutic target.

## 2. Materials and Methods

We conducted a narrative review via the following steps: the formulation of the research question; a database query for relevant studies; iterative, team-based article selection; data analysis; and results summarization and discussion.

The PubMed database was queried using the keywords “Th9” and “Interleukin 9” and “Airway inflammation” and “Allergic Rhinitis” and “Chronic Rhinosinusitis”, resulting in 54 manuscripts between 2005 and 2025. Restricting the search to free, full-text articles yielded 38 results. Of these, 30 articles were published in Medline and only 23 in English. The search results were imported into an online cloud database for further analysis.

An analysis of the included articles was carried out independently by two groups of three reviewers (M.D., O.B., and D.V.; G.M., A.O., and A.Z.C.A.), who reviewed the titles, abstracts, and full texts. Any discrepancies were resolved by C.S., who is not a clinical airway specialist but a molecular biology specialist. In this way, we hoped to minimize and prevent possible bias.

## 3. Allergic Rhinitis

Nouri-Aria, Pillete, Jacobson, Watanabe, and Durham conducted a study involving 44 patients with severe summer seasonal allergic rhinitis, recruited from the Royal Brompton Hospital in London, UK. These patients exhibited uncontrolled symptoms despite antiallergic medication and had positive skin-test responses to timothy grass pollen. The study analyzed participants before and two years after undergoing a double-blind trial of grass pollen immunotherapy. Dual immunofluorescence identified cells expressing IL-9 protein, in situ hybridization detected IL-9 mRNA-positive cells, and sequential immunochemistry and in situ hybridization identified cell phenotypes. The results showed the upregulation of IL-9 in the nasal mucosa during the pollen season, correlating with eosinophil infiltration. Successful pollen immunotherapy significantly reduced IL-9 mRNA-positive cells from nonendothelial sources, with IL-9 primarily derived from T cells, eosinophils, neutrophils, and mast cells [11].

At Vrje Universiteit Brussel, 30 adult volunteers (17 women and 13 men) with allergic complaints (asthma: 4; rhinitis/conjunctivitis: 9) were studied for atopy based on allergen-specific IgE using RAST (UniCAP fluoroenzymeimmunoassays), and a second group of 24 atopic subjects (17 women and 7 men) with allergies to birch or Dermatophagoides pteronyssinus (Der p) was recruited from the Pneumology Unit at Cliniques Universitaires Saint-Luc. Peripheral blood mononuclear cells (PBMCs) from these subjects were cultured with allergens or phytohemagglutinin (PHA), and cytokine levels (IL-5, IL-9, IL-13) were measured using the ELISA. IL-9 production was observed in response to Der p allergens in hypersensitive adults, comparable to PHA-induced levels. IL-9, IL-5, and IL-13 production was higher in atopic individuals than that in nonatopic controls. Significant correlations were found between the IL-9 levels and allergen-specific IgE for Der p and Bet v 1 (birch allergen) peptides [12].

Hans Peter Huaber and colleagues investigated IL-9 expression and its role in inducing chloride channel hCLCA1 in the nasal mucosa of allergic patients after localized allergen challenges. Fourteen patients with seasonal allergic rhinitis were recruited. Baseline biopsies were collected during the off-season, and follow-up biopsies were taken 24 h after allergen or saline nasal spray challenges. The results revealed IL-9 immunoreactivity in submucosal inflammatory cells and hCLCA1 expression in mucus-producing epithelial cells. Ragweed allergen exposure caused significant increases in eosinophils, IL-9-positive cells, and hCLCA1-positive epithelial cells, with no notable changes in T cells [13].

Manel Jordana and colleagues at McMaster University, Canada, recruited 20 patients with seasonal rhinitis and 15 controls before the ragweed pollen season in Ontario. PBMCs from these subjects spontaneously expressed low or undetectable IL-4, IL-5, IL-9, IL-10, and IL-13 mRNA levels. Upon ragweed stimulation, allergic patients showed the significant upregulation of these cytokines, with IL-9 gene expression peaking between 12 and 72 h. Spontaneous IL-9 protein production was similar between controls and patients; however, adding exogenous IL-10 reduced IL-9 production in allergic patients [14].

In northern Italy (Genoa and Pavia), researchers studied 35 patients with allergic rhinitis (AR) monosensitized to Parietaria during peak pollen season and 38 AR patients monosensitized to birch after their pollen season ended. The median serum IL-9 level in Parietaria-allergic patients was 12.6 pg/mL (range: 5.03–24.11), significantly higher than that in birch-allergic patients (4.83 pg/mL, range: 1.97–14.01; *p* < 0.05). Parietaria-allergic patients exposed to allergens also exhibited higher IL-9 levels than those of birch-allergic patients not exposed to allergens (*p* < 0.05) [15].

Ciprandi, De Amici, and Marseglia conducted a study to determine whether sublingual immunotherapy (SLIT) could modulate IL-9 serum levels in patients with persistent allergic rhinitis (AR) caused by Parietaria allergy. The study evaluated 21 AR patients successfully treated with SLIT and 52 AR patients managed solely with medication during the pollen season. The results showed significantly lower serum IL-9 levels in SLIT-treated patients compared with those in untreated patients (*p* < 0.0001) [16].

G.W. Scadding carried out research at the Royal Brompton Hospital allergy clinic, comparing nasal and skin responses between untreated individuals with seasonal grass pollen allergic rhinitis, those with immunotherapy-treated allergics, and a nonatopic control group. Patients treated with immunotherapy exhibited lower symptom scores and higher peak nasal inspiratory flows (PNIFs) after the allergen challenge compared with untreated patients. Immunotherapy also led to reduced IL-4, IL-9, and eotaxin concentrations in nasal fluid, along with trends indicating lower IL-1 and early-phase tryptase levels. Notably, the nasal fluid IL-4 and IL-9 levels were significantly lower in immunotherapy-treated patients at 8 h post-challenge [17].

At the First Affiliated Hospital of Chongqing Medical University in China, researchers assessed Th9 cells and associated factors by measuring interleukin-9 (IL-9), PU.1, interferon-regulatory factor 4 (IRF4), and Th9 cell numbers. The study enrolled 30 AR patients and 20 healthy controls between January and April 2014. AR patients exhibited significantly higher IL-9, PU.1, IRF4, and Th9 cell levels. Positive correlations were observed between the IL-9 levels and disease severity markers, including EOS expression, RQLQ, and VAS scores. Spearman’s correlation analysis further demonstrated significant positive relationships between the IL-9 protein levels, IL-9 mRNA expression, Th9 cell frequency, and AR severity [18].

Fatahi F conducted a case–control study between 2009 and 2010 at Kashani Hospital in Shahrekord, Iran, to investigate gene polymorphisms in women with AR. The study involved 195 nonasthmatic AR patients and 199 healthy controls from the Fars ethnic group. SNPs from IL9, IL9R, IL17A, and IL17F were genotyped. The rs731476 SNP in IL9R showed significantly different allelic and genotypic distributions between AR patients and controls, with the rs731476 T-/rs2069885 G genotype conferring a higher risk of AR development in the gene–gene interaction analysis [19].

Ali Askari and colleagues evaluated the effects of fexofenadine (120 mg/day) and fluticasone propionate (100 µg/day) on the Th9 cell differentiation and gene expression of transcription factors, including IRF4, BATF, and PU.1. The study involved 26 AR patients treated at Dr. Mohammad Kermanshahi Hospital between July and October 2019. Peripheral blood samples collected before and after one month of treatment revealed significant reductions in the IRF4 and BATF expression levels, which were associated with an observed reduction in the AR symptom severity. However, the PU.1 gene expression remained unchanged before and after treatment [20].

The study conducted by Lihua Li from the Department of Otorhinolaryngology Head and Neck Surgery, The Second Affiliated Hospital of Nanchang University, Nanchang, China, found that long noncoding RNA HOTAIRM1 is highly expressed in AR. HOTAIRM1 promotes Th9 cell differentiation and AR progression by binding to miR-148a-3p and upregulating IRF4. In turn, IRF4 activates HOTAIRM1 transcription, forming a positive-feedback loop. Silencing HOTAIRM1 reduced AR symptoms, IgE and IL-9 levels, and Th9 cells. Thus, HOTAIRM1 contributes to AR development through the HOTAIRM1/miR-148a-3p/IRF4 axis and may be a potential therapeutic target [21].

Palomares et al. conducted a 3-day nasal allergen challenge (NAC) with Dermatophagoides pteronyssinus extract (100 HEP/mL, Leti Pharma, Barcelona, Spain) in nine patients with local allergic rhinitis (LAR), five with allergic rhinitis (AR), and five healthy controls (HCs). NAC-DP significantly increased the peripheral IL-9^+^ Th9 cells in LAR patients but not in AR patients or HCs. In contrast, IL-4^+^ Th2 cells rose similarly in the LAR and AR groups but not in the HCs. Upon allergen stimulation, IL-4^+^ Th2 cell proliferation was greater in AR patients than that in LAR patients and HCs, while IL-9^+^ Th9 cell proliferation was the highest in LAR patients compared with AR patients and HCs. Moreover, IL-4 levels were elevated in AR patients, whereas IL-9 levels were higher in LAR patients than those in AR patients and HCs [22].

Liu et al. investigated the relationship between IL-9, Th9 cells, and BAFF levels in the peripheral blood of patients with allergic rhinitis (AR) and their association with disease severity. The retrospective study included 80 AR patients (divided into mild and moderate-to-severe groups) and 50 non-AR controls undergoing nasal surgeries.

The results show that the IL-9, Th9, and BAFF levels were significantly higher in AR patients than in controls (*p* < 0.05), with the highest levels in the moderate-to-severe group. A multivariate logistic regression identified IL-9, Th9, and BAFF as independent risk factors for moderate-to-severe AR. An ROC curve analysis revealed good diagnostic accuracy for IL-9 (AUC = 0.770), Th9 (AUC = 0.734), and BAFF (AUC = 0.761), while the combined detection achieved a higher AUC of 0.888, indicating a superior predictive value [23].

**Figure 2:**
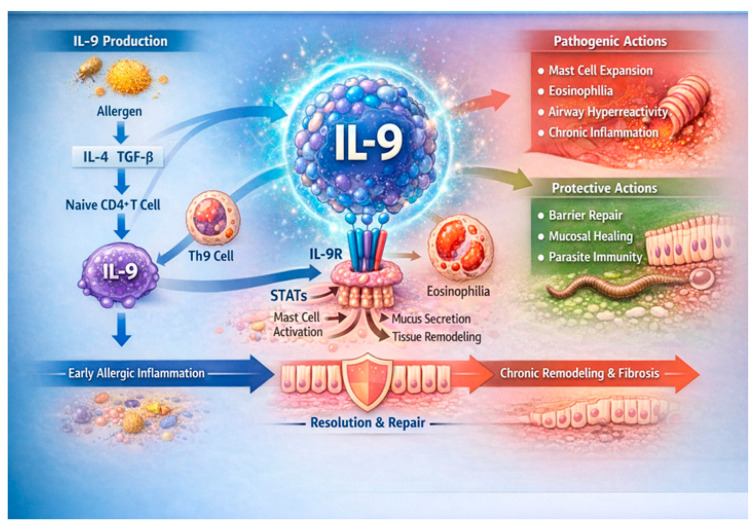
Chart depicting molecular interplay of IL-9/Th9 axis in allergic rhinitis. Allergen-induced IL-4 and TGF-β drive differentiation of naïve CD4^+^ T cells into Th9 cells, which secrete IL-9. IL-9 signals through IL-9R and downstream STAT pathways in mast cells, eosinophils, epithelial cells, and fibroblasts, promoting mucus production, airway hyperreactivity, and type-2 inflammation. Concurrently, IL-9 supports epithelial barrier repair and mucosal healing, influencing progression toward resolution or chronic airway remodeling. IL-9—interleukin-9; Th9—T helper 9 cell; IL-4—interleukin-4; TGF-β—transforming growth factor-β; CD4^+^—cluster of differentiation 4 positive; IL-9R—interleukin-9 receptor; STAT—signal transducer and activator of transcription.

There is increasing interest in decrypting the role of Th9 and IL9 in the mechanisms of allergic rhinitis (Figure 2).

## 4. Rhinosinusitis

Dong Lin from the Department of Biology and Chemical Engineering, Fuqing Branch of Fujian Normal University, Fujian, China, conducted a study on 67 chronic rhinosinusitis (CRS) patients, including 35 with CRS with nasal polyps (CRSwNPs), 32 with CRS without nasal polyps (CRSsNPs), and 20 control subjects. The study examined the IL-9 expression and regulation in CRS using immunohistochemistry, Western blotting, and real-time polymerase chain reaction to assess the IL-9 and IL-9R immunolabeling, protein, and mRNA levels. IL-9 and IL-9R were significantly overexpressed in CRS patients compared with controls, primarily localized in nasal mucosal epithelial, submucosal inflammatory, and gland epithelial cells. IL-4, IL-17A, IL-1β, and IL-4 collectively contributed to increased IL-9 levels, suggesting that IL-9 plays a role in epithelial and inflammatory cell proliferation in CRS [24].

Olcott, Han, Cunningham, and Franzese from the Eastern Virginia Medical School (EVMS) studied 54 patients (36 CRSwNP patients, 9 CRSsNP patients, and 9 controls). Nasal polyp and ethmoid sinus mucosal samples were evaluated via immunohistochemistry for IL-9 and IL-17C expression in submucosal inflammatory cells. The IL-9 and IL-17C expression was significantly higher in CRS patients than that in controls. While the IL-9 difference between CRSwNP and CRSsNP patients was not statistically significant, CRSwNP patients with atopy exhibited higher IL-9 expression [25].

A single-center prospective study at Service d’ORL et de chirurgie cervico-faciale, Hôpital Huriez, CHRU, Lille, France, included 57 CRSwNP patients over 18 months. Fifteen had allergies, with house dust mites, grass pollen, and cat dander being the most common allergens. The difference in the nasal secretion IL-9 levels between the allergic patients (28.0 pg/mL) and controls (35 pg/mL) was not significant [26].

A study using liquid chromatography–mass spectrometry (LC-MS/MS) in data-dependent (DDA) and data-independent acquisition (DIA) modes analyzed CRS and nasal polyps. Nasal secretions from 10 controls, 10 CRSsNP patients, and 10 CRSwNP patients were collected using filter paper placed on the middle meatus for 10 min. A total of 2020 proteins were identified, with the IL-7, IL-9, IL-17A, and IL-22 signaling pathways significantly elevated in CRSwNP, indicating increased neutrophil activation and degranulation [27].

Delemarre and colleagues characterized the IL-9 and IL-9R expression in CRSwNP tissues and investigated Staphylococcus aureus as a local IL-9 trigger. The IL-9 and IL-9R gene expressions were significantly elevated in CRSwNP patients compared with those in controls, particularly in CRSwNP patients with comorbid asthma. IL-9+ cells were predominantly mononuclear cells and certain neutrophils, suggesting subtype- and microenvironment-dependent activation. The IL-9 expression in peripheral blood mononuclear cells (PBMCs) was significantly increased upon stimulation with S. aureus and staphylococcal enterotoxin B (SEB), but not with S. epidermidis or LPS [28].

A prospective olfactory outcome study enrolled 62 CRS patients (≥18 years) from rhinology clinics at the Medical University of South Carolina, Oregon Health and Science University, University of Utah, University of Virginia, and University of Colorado. The IL-9 levels in olfactory cleft mucus ranged from 2.6 to 764.7 ng/mL (mean: 41.3 ng/mL) and were higher in CRSwNP than those in CRSsNP. IL-9 levels correlated with worse olfaction in CRSwNP (*p* = 0.05) but not in CRSsNP (*p* = 0.570) [29].

Between 2016 and 2021, a multi-institutional observational study prospectively recruited 127 CRS patients (53 CRSsNP patients; 74 CRSwNP patients) to examine their mucus cytokine levels and the correlation of the mucus cytokine levels with the Sino-Nasal Outcome Test (SNOT-22). Mucus from the olfactory cleft was analyzed for cytokine biomarkers. IL-9 was the only biomarker significantly correlated with the SNOT-22 total scores, rhinology subdomain scores, Lund–Mackay (LM) scores, and patient-reported sense of smell [30].

**Figure 3:**
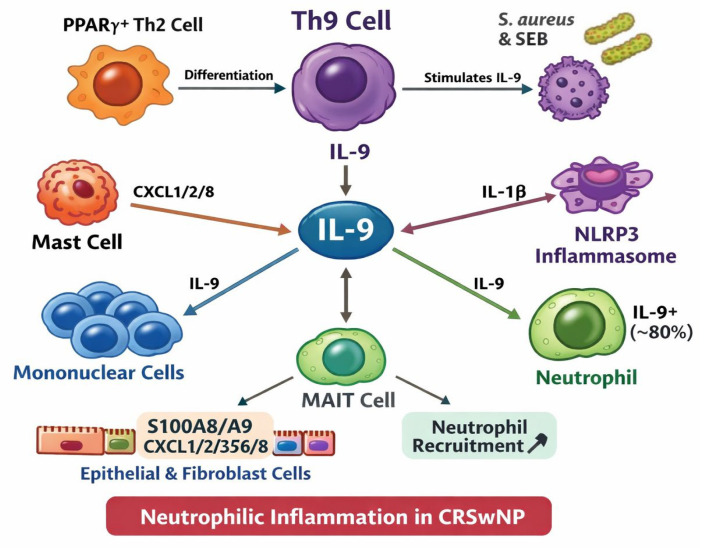
Chart depicting molecular interplay of IL-9 in chronic rhinosinusitis. Th9 cells, induced by *S. aureus* and SEB, produce IL-9, which acts on mononuclear cells, MAIT cells, and IL-9^+^ neutrophils (~80%). IL-9 promotes neutrophil recruitment through epithelial and fibroblast release of S100A8/A9 and CXCL1/2/3/5/8, further amplified by mast cell-derived CXCL1/2/8. NLRP3 inflammasome activation and IL-1β reinforce neutrophilic inflammatory cascade. CRSwNP—chronic rhinosinusitis with nasal polyps; SEB—staphylococcal enterotoxin B; Th—T helper cell; PPARγ—peroxisome proliferator-activated receptor-γ; MAIT—mucosal-associated invariant T-cell; CXCL—C-X-C motif chemokine ligand; NLRP3—NOD-like receptor family pyrin domain-containing 3.

Figure 3 underlines the progression of the research in the last 10 years regarding the connection between Th9 and IL9 in chronic rhinosinusitis.

## 5. Genetic Studies

The study conducted by Kadhom et al. evaluated the association between IL-5 (rs2069812T/C) and IL-9 (rs1859430C/T) gene polymorphisms and the risk of allergic asthma in Iraqi patients. A total of 240 subjects (120 patients and 120 healthy controls) were analyzed. IL-5 and IL-9 serum levels were measured by ELISA, and polymorphisms were detected using allele-specific PCR. Patients with allergic asthma showed significantly higher IL-5 and IL-9 levels than those of controls (*p* < 0.05). No significant differences were found in age, gender, or residence between groups. The TG genotype was more frequent among patients (66 vs. 58) and was associated with a higher asthma risk (OR = 2.41, 95% CI: 1.25–4.65). The GG genotype also appeared more often in patients (65 vs. 54) and was identified as a minor risk factor (OR = 1.06, 95% CI: 0.64–1.75) [31]. In China, L.X.Chen et al. investigated the relationship between IL-18 and IL-9 gene polymorphisms and asthma susceptibility, as well as their association with cytokine expression levels. A total of 200 asthma patients and 200 healthy controls were analyzed. DNA sequencing identified IL-18 and IL-9 variants, while the ELISA was used to measure the serum cytokine levels. The results showed no significant differences in IL-18 (rs189667, rs360715) alleles between groups but significant differences for IL-9 (rs1859430, rs2066758) polymorphisms (*p* = 0.001, *p* = 0.022). The G and T alleles were more frequent in asthma patients. The GG genotype at rs1859430 was higher in patients (*p* = 0.005), while the CC genotype at rs2066758 was lower (*p* = 0.044). Haplotype analysis revealed increased GT and decreased AT frequencies in patients (*p* = 0.000, *p* = 0.006). Patients with TT (rs360715) had higher IL-18 levels, and those with AG (rs1859430) had higher IL-9 levels (*p* < 0.05). Genotypes CT (rs360715) and CC (rs2066758) were linked to altered PaO_2_ and PaCO_2_, respectively [32].

Mahdaviani et al. conducted one case–control study on 70 asthmatic patients and 77 nonasthmatic healthy controls aged 18–60 who were referred to “Masih Daneshvari Hospital” in Tehran, Iran. Asthmatic subjects were classified into three groups: mild persistent, moderate persistent, and severe persistent. IL9 promoter rs2069882 polymorphisms were genotyped in all cases and the control group. All the SNPs were in accordance with Hardy–Weinberg equilibrium in both groups. IL9 rs2069882 SNPs were not significantly correlated with asthma susceptibility [33]. Fatahi et al. assessed the association of the single-nucleotide polymorphisms (SNPs) of IL9, IL9R, IL17A, and IL17F and the potential interaction of these genes with the determination of IgE levels in women with allergic rhinitis (AR) in Shahrekord, Iran. SNPs from IL9, IL9R, IL17A, and IL17F were genotyped in 394 random samples, including 195 AR patients and 199 normal controls in a case–control study. In the gene–gene interaction analysis, IL9R/IL9 genotype rs731476 T-/rs2069885 G conferred a higher risk for AR phenotype development [19].

**Figure 4:**
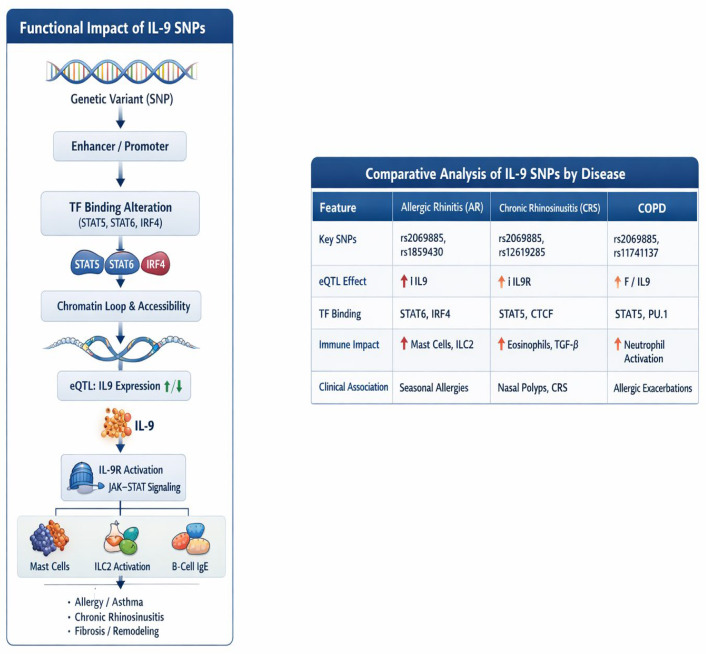
**Functional impact of IL-9 SNPs and disease-specific associations.** IL-9-related single-nucleotide polymorphisms (SNPs) modify transcription factor (TF) binding, chromatin accessibility, and *IL9*/*IL9R* expression, shaping downstream Janus kinase–signal transducer and activator of transcription (JAK–STAT) signaling and immune activation. Comparative profiles highlight distinct SNP patterns, expression quantitative trait locus (eQTL) effects, and immune signatures across allergic rhinitis (AR), chronic rhinosinusitis (CRS), and chronic obstructive pulmonary disease (COPD).

Figure 4 summarizes the core data from these studies in a graphical manner.

## 6. Animal Studies

Even though there are many human studies on interleukin-9 (IL-9) and T helper 9 cells (Th9), animal studies still add unique types of evidence that human research usually cannot provide. This is not redundancy—it is about depth, control, and causality. Animal studies were included because they provide insights that human studies usually cannot via proven causation (by manipulating interleukin-9 or T helper 9 cells, researchers can show direct effects, not just associations), controlled conditions (animals allow uniform genetics and environments, reducing confounding factors), access to tissues (researchers can study multiple organs and early disease stages in detail), safe treatment testing (new therapies targeting IL-9/Th9 can be evaluated before human trials), and disease development tracking (animal models show how diseases start and progress over time), [34,35,36,37].

Gu, Wang, and Cao investigated the role of Th9 cells in allergic rhinitis (AR) using murine models. Eight-week-old female BALB/c mice were assigned to four groups (10 mice per group): (1) a control group (group A)—sensitized and challenged with saline; (2) an OVA group (group B)—sensitized and challenged with ovalbumin (OVA); (3) an isotype group (group C)—treated with isotype antibodies (Abs) for anti-IL-9; and (4) an anti-IL-9 group (group D)—treated with anti-IL-9 Ab. Mice in groups C and D received intranasal instillations of either hamster IgG (isotype Ab for anti-IL-9) or anti-IL-9 Abs 30 min before the OVA challenge, and control-group mice received saline instead of OVA. The study assessed AR symptoms by counting sneezes and nasal rubs post-challenge. The mice in group B exhibited significantly more sneezing than those in group A, while the anti-IL-9 treatment in group D reduced sneezing compared with that in group C. The IL-9 levels in the nasal mucosa were significantly elevated in the AR group (group B) compared with those in the control group (group A) and were reduced in the anti-IL-9-treated mice (group D) compared with the isotype-treated controls (group C), measuring 19.8 ± 3.0 pg/mL (group A), 65.3 ± 5.2 pg/mL (group B), 63.4 ± 3.8 pg/mL (group C), and 36.3 ± 2.0 pg/mL (group D). The anti-IL-9 treatment suppressed nasal mucosa inflammation by reducing multiple inflammatory mediators. The relative Il-9 mRNA expression was significantly decreased in group D compared with that in group C, with values of 1.03 ± 0.26 (group A), 20.10 ± 1.18 (group B), 19.50 ± 1.73 (group C), and 5.57 ± 0.46 (group D) [38].

Soo Whan Kim et al. studied the role of IL-9 antibodies in inducing tolerance in mice. Forty mice were divided into four groups: a control group (n = 10), an AR group (n = 10), an oral tolerance (OT) group (n = 10), and an OT with anti-IL-9 Ab (OT+IL9AB) group (n = 10). Sneezing and nose-rubbing motions were recorded 15 min after the final allergen challenge. The OT and OT+IL9AB groups exhibited fewer sneezing motions than the AR group, with the OT+IL9AB group showing a significant reduction compared with the OT group. The numbers of nasal rubs were higher in the OT and OT+IL9AB groups than those in the AR group, while serum OVA-specific IgE levels were significantly elevated in the AR group compared with the OT and OT+IL9AB groups, with a significantly lower level in the OT+IL9AB group than that in the OT group. Eosinophil counts were higher in the AR group than those in the OT and OT+IL9AB groups, with the OT+IL9AB group having a lower count than that of the OT group. PU.1 Moreover, mRNA expression was higher in the AR group compared with that in the OT and OT+IL9AB groups, with the OT+IL9AB group exhibiting significantly lower expression than that of the OT group. Anti-IL-9 antibodies reduced allergic inflammation by suppressing Th2 and Th17 cells, while enhancing regulatory T-cell-mediated tolerance [39].

Porcelain berry (*Ampelopsis glandulosa*), a wild grape species native to East Asia, has reported anti-inflammatory, anti-hepatotoxic, and anti-osteoclastogenesis properties. Awa tea, a traditional fermented tea from Tokushima, contains Lactobacillus pentosus and Lactobacillus plantarum and has demonstrated anti-obesity effects, the suppression of mono- and disaccharide-induced blood glucose increases, and potential allergic disease treatment benefits. Hiroyuki Miziguchi and colleagues examined the impact of wild grape hot water extract (WGE) on PKCδ-mediated H1R and NFAT-mediated IL-9 gene expressions, which contribute to acute allergic rhinitis symptoms in TDI-sensitized rats. Six-week-old male Brown Norway rats were divided into seven groups (five rats per group), including a control group, a TDI-sensitized group, and test groups. TDI sensitization increased the H1R and IL-9 mRNA expression in the nasal mucosa of TDI-sensitized rats, and ionomycin-induced IL-9 gene upregulation in RBL-2H3 cells was suppressed in a manner that depended on the Awa tea dose. The combination of WGE and Awa tea significantly suppressed H1R and IL-9 gene upregulation more effectively than Awa tea alone [40].

Chunping Yang et al. conducted research on Th9 activation in allergic rhinitis using a murine model. Male BALB/c mice were sensitized via an intraperitoneal ovalbumin injection. The study included four groups (N = 8): a control group, an AR group, an IL-9 shRNA + AR group, and a vector + AR group. The IL-9 levels were significantly higher in the AR group compared with those in the control, and the Th2, Th9, Th17, and Treg cell ratios were also significantly elevated in the AR group but were reduced following IL-9 shRNA silencing. These findings suggest that Th9 activation plays a critical role in allergic rhinitis pathogenesis, which can be mitigated by IL-9 suppression [41].

Six-week-old female BALB/c mice were used to investigate the suppressive effects of dexamethasone on the Th9 cell involvement in allergic rhinitis pathogenesis. BALB/c mice, transferred with in vitro-differentiated Th9 cells, were subcutaneously administered dexamethasone and subsequently challenged with an intranasal injection of ovalbumin (OVA). A flow cytometry analysis revealed a significant increase in infused Th9 cells within the nasal-associated lymphoid tissue (NALT) following allergen exposure, as indicated via DO11.10-TCR detection. Dexamethasone administration significantly suppressed allergen-induced nasal hyperresponsiveness, eosinophil migration in Th9 cell-transferred mice, and the overall allergen-induced Th9 cell migration [42].

Zhaowei Gou and colleagues examined the effects of IL-9-neutralizing antibodies in eighty female BALB/c mice (aged 6–8 weeks), randomly divided into four groups (20 per group): an allergic rhinitis group, a control group, an IL-9-neutralizing-antibody-treated group, and a homotypic control group. OVA was used as the sensitizing agent. Treatment with IL-9-neutralizing antibodies led to a reduction in sneezing, nasal scratching, and OVA-IgE levels in peripheral blood in the allergic rhinitis group. Additionally, eosinophil and mast cell infiltration in the nasal mucosa was significantly inhibited. A flow cytometry analysis revealed a reduction in Th2 cells within the spleen following the IL-9-neutralizing antibody treatment. Moreover, the TSLP, TSLPR, OX40, OX40L, and IL-7R mRNA expression levels were significantly decreased in the allergic rhinitis group after treatment. These findings suggest that IL-9-neutralizing antibodies may exert therapeutic effects in allergic rhinitis, potentially through modulation of the TSLP-OX40/OX40L signaling and JAK1/2-STAT5 pathways [43].

Recent studies have implicated IL-9, pyroptosis, serum- and glucocorticoid-induced protein kinase 1 (SGK1), NOD-like receptor 3 (NLRP3), and nuclear factor kappa-B (NF-κB) in the development of allergic rhinitis. To explore this further, forty-five female BALB/c mice were randomly assigned to a control group, an allergic rhinitis group, and an IL-9-neutralizing-antibody-treated group. Treatment with IL-9-neutralizing antibodies significantly inhibited sneezing and nasal scratching in allergic rhinitis mice compared with controls, and microscopic analysis confirmed that eosinophil and mast cell infiltration was effectively suppressed. Furthermore, IL-9-neutralizing antibody treatment led to a decrease in p-p65 protein levels in allergic rhinitis while increasing SGK1 mRNA and protein levels. IL-9 levels in the nasal mucosa were also reduced following IL-9-neutralizing antibody treatment. An immunohistochemical analysis demonstrated the decreased expression of pyroptosis-related markers in the nasal mucosa after treatment, including Caspase-1, ASC, and NLRP3. Additionally, the mRNA levels of Caspase-1, ASC, and NLRP3 were significantly reduced. These findings suggest that IL-9 may contribute to the pathogenesis of allergic rhinitis through the SGK1 and NF-κB/NLRP3/ASC signaling pathways [44].

IL-9/Th9 signatures are more consistent and directionally aligned with mast cell-rich, type-2-skewed AR/CRS/heavy T2 endotypes than with neutrophilic endotypes; however, they are not currently better standalone markers than classic/established type-2 markers (e.g., IL-5, IL-13, IgE, tissue eosinophils, mast cell mediators) (Table 1).

## 7. Cross-Disease Comparative Analysis Between Allergic Rhinitis, CRS, Asthma, and COPD

**Figure 5:**
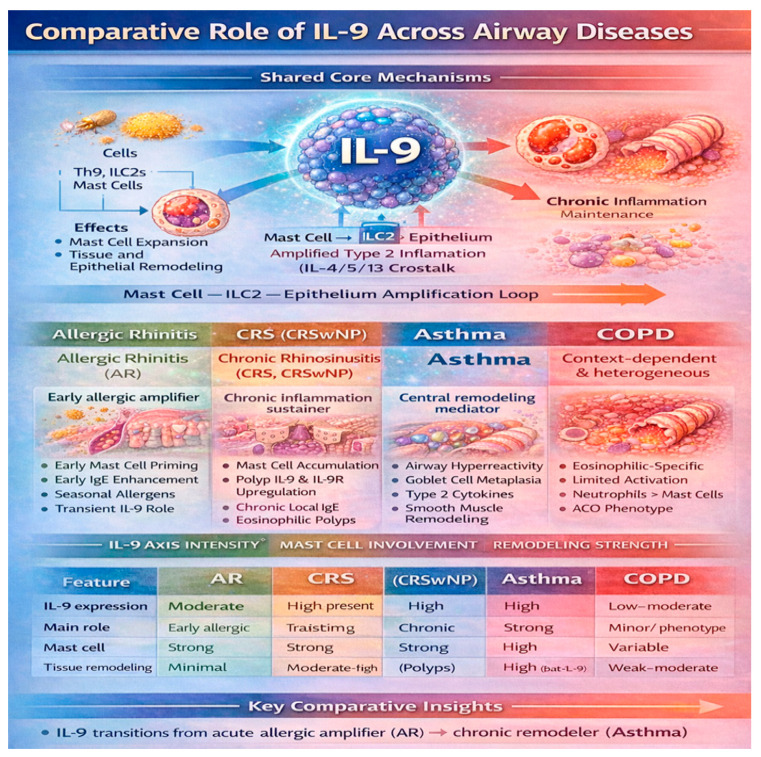
Comparison of IL-9 roles across airway diseases. IL-9 drives shared type-2 inflammatory pathways—including mast cell expansion, ILC2 activation, epithelial remodeling, and Th9-mediated amplification—while exhibiting disease-specific patterns across allergic rhinitis (AR), chronic rhinosinusitis (CRS), chronic rhinosinusitis with nasal polyps (CRSwNP), asthma, and chronic obstructive pulmonary disease (COPD). The comparative matrix highlights differences in the IL-9 expression, mast cell involvement, and remodeling intensity across airway disease phenotypes.

In the previous paragraphs, we described the roles of IL9 and Th9 in allergic rhinitis and rhinosinusitis. Th9 and IL9 are players in lung inflammation, and asthma represents the condition in which IL-9 exerts important biological effects. IL-9 contributes to airway hyperresponsiveness, goblet cell metaplasia, mucus hypersecretion, and mast cell infiltration into airway smooth muscle. Experimental models have demonstrated that IL-9 deficiency attenuates airway inflammation and structural remodeling, underscoring its importance in disease progression [45]. Nevertheless, clinical trials targeting IL-9 have shown limited efficacy, likely due to redundancy within the type-2 cytokine network. In COPD, IL-9 plays a comparatively minor and heterogeneous role and is primarily associated with eosinophilic COPD phenotypes and asthma–COPD overlap syndromes. In contrast, the predominant inflammatory pathways in COPD are driven by neutrophilic and type-1/type-17 immune responses, which diminishes the relative contribution of IL-9 [3]. A comparative analysis reveals three principal gradients in IL-9 involvement across airway diseases. First, IL-9 expression transitions from transient in AR to persistent in CRS and asthma, reflecting increasing disease chronicity. Second, the contribution of IL-9 to tissue remodeling is minimal in AR, moderate in CRS, and the most pronounced in asthma, while it is largely independent in COPD. Third, IL-9 activity closely parallels type-2 inflammatory dominance, which is strong in AR, CRS, and asthma but reduced and heterogeneous in COPD. These observations support the concept of a unified airway model in which IL-9 acts as a vertical integrator linking upper and lower airway inflammation. Within this framework, IL-9 contributes to disease initiation in the upper airway and to amplification and remodeling in the lower airway, with a diminishing influence in distal airway disease (Figure 5).

## 8. Future Directions and Limitations

**Inflammation**

A future direction must be the translation of these findings into clinical practice, either by developing more specific diagnostic tests for allergies and chronic inflammatory diseases or through the development of novel biologic drugs for autoimmune diseases [46].

Piezo1 influences the mitochondrial metabolic pathway during Th9 cell differentiation primarily by regulating the SIRT3–SDHA–OXPHOS axis. The activation of Piezo1 promotes the expression and activity of SIRT3, a mitochondrial deacetylase, which, in turn, enhances the activity of SDHA, a key component of mitochondrial respiratory chain complex II. This activation boosts oxidative phosphorylation (OXPHOS), providing the energy required for Th9 cell differentiation. Additionally, Piezo1 activation leads to the translocation of HIF1α from the cytoplasm to the nucleus, further promoting the differentiation process. Conversely, inhibiting Piezo1 suppresses this pathway, reducing OXPHOS activity and impairing Th9 cell development [47].

Researchers have managed to investigate the anticancer properties of Th9 cells by using a combination of in vitro and in vivo experiments, including the adoptive transfer of Th9 cells into mice bearing tumors in the lungs. Th9 cells possess a superior ability to migrate to the lungs and eliminate TNBCs and OS cells developing there, and this process is mediated by the CXCL12–CXCR4 pathway. The study has several limitations, including the use of mouse models, which may not fully replicate the human disease, and the need for further investigation into the mechanisms underlying the anticancer properties of Th9 cells [48].

The expression of IL-9 and its associated transcription factors is elevated in patients with autoimmune diseases, and Th9 cell functions are regulated by various factors, including extracellular signaling pathways, transmembrane proteins, intracellular transcription factors, metabolic components, and pathogens. Th9 cells play a pivotal role in inflammatory immune responses, and targeting Th9 cells may provide a promising avenue for the development of novel therapies to prevent or treat autoimmune diseases. Most of the current functional studies were conducted on animal models, whereas data on IL-9 expression, transcription factor profiles, and the Th9 cell distribution in human autoimmune diseases are primarily derived from case–control studies. These results indicate that Th9 cells are involved in inflammatory bowel disease (IBD) pathogenesis. Targeting Th9 cells may provide a promising avenue for the development of novel therapies to prevent or treat autoimmune diseases [49].

A recent study used quantitative PCR (qPCR) to quantify the expression levels of Th9-associated genes, and flow cytometry was also used to analyze the frequency of Th9 and SUCNR1+ CD4+ T cells. Succinate levels were elevated in the fecal samples of Oxa-colitis mice compared with those in controls, and treatment with a SUCNR1 antagonist or an anti-IL-9 antibody alleviated colonic inflammation. The study found that succinate levels are elevated in colitis mice and UC patients, and that blocking the succinate/SUCNR1 axis reduces intestinal inflammation in mice. Colonic microbiota triggers succinate-mediated pro-inflammatory responses in oxazolone colitis. Additionally, SUCNR1 antagonist 7a inhibits Th9 cell differentiation in a dose-dependent manner [50].

According to a study by Jiajie Tu et al., rheumatoid arthritis is encouraged to develop via the positive-feedback loop of Th9 cells, namely, PU.1–IL9. Th9 cells are well known for producing interleukin-9 (IL-9), which has been linked to a number of autoimmune disorders, and for expressing the transcription factor PU.1. However, more research is necessary to determine its exact connection to the pathophysiology of rheumatoid arthritis. ELISA, Western blotting (WB), and immunohistochemistry staining were used in the study to measure the PU.1 and IL-9 expression levels in RA patients. Transcription, and Th9-derived IL-9, triggers PU.1 through the IL-9R-JAK1/STAT3 pathway. These findings provide evidence that the Th9 deregulation of RA involves a positive loop formed by the PU.1–IL-9 axis [51].

**IL-9 as a Potential Bridge Between Type-2 and Neutrophilic Responses**

**Figure 6:**
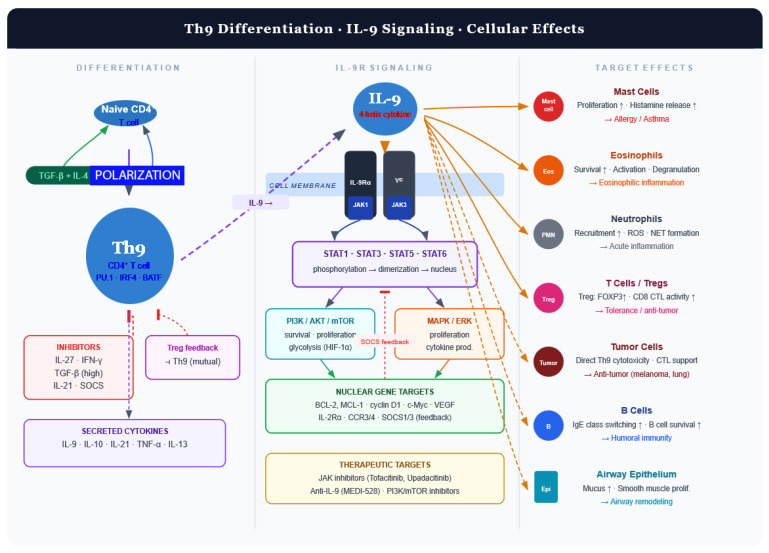
Mechanistic framework of Th9–IL-9 axis. Th9 cells differentiate from CD4^+^ T cells under TGF-β and IL-4 and secrete IL-9. IL-9 signals through IL-9R to activate JAK–STAT and PI3K/ERK pathways, promoting cell survival and proliferation. IL-9 acts on multiple immune and structural cells, contributing to inflammation, asthma, and anti-tumor immunity. Th9—T helper 9 cell; IL-9—interleukin-9; IL-9R—IL-9 receptor; TGF-β—transforming growth factor-β; PU.1, IRF4, BATF—Th9-associated transcription factors; JAK—Janus kinase; STAT—signal transducer and activator of transcription; PI3K—phosphoinositide 3-kinase; AKT—protein kinase B; mTOR—mechanistic target of rapamycin; MAPK/ERK—mitogen-activated protein kinase/extracellular signal-regulated kinase; HIF-1α—hypoxia-inducible factor-1 alpha; BCL-2, MCL-1—anti-apoptotic proteins; CTL—cytotoxic T lymphocyte; Treg—regulatory T-cell; ROS—reactive oxygen species; NETs—neutrophil extracellular traps.

Several mechanisms from the broader literature suggest how IL-9 could connect T2 and neutrophilic CRS: IL-9 activates mast cells, which release CXCL1/2/8, key chemokines driving neutrophil recruitment in CRSwNP [52]. The NLRP3 inflammasome integrates signals from LPS, poly I:C, and IL-4 to drive IL-1β production and neutrophil recruitment. IL-1β induces epithelial and fibroblast subsets that release S100A8/A9 and CXCL1/2/3/5/6/8, directly promoting neutrophil infiltration. Because IL-9 can induce IL-1β from mast cells, it may feed into these pathways [53,54]. MAIT cells with type-17 features are enriched in neutrophilic or mixed polyps and promote neutrophil recruitment; IL-9 can modulate innate-like T cells, although a direct IL-9–MAIT axis in CRS remains unproven [55]. We have compiled a mechanistic framework for Th9 differentiation pathways, IL-9R signaling, and downstream cellular effects in Figure 6.

IL-9–mast cell crosstalk

**Figure 7:**
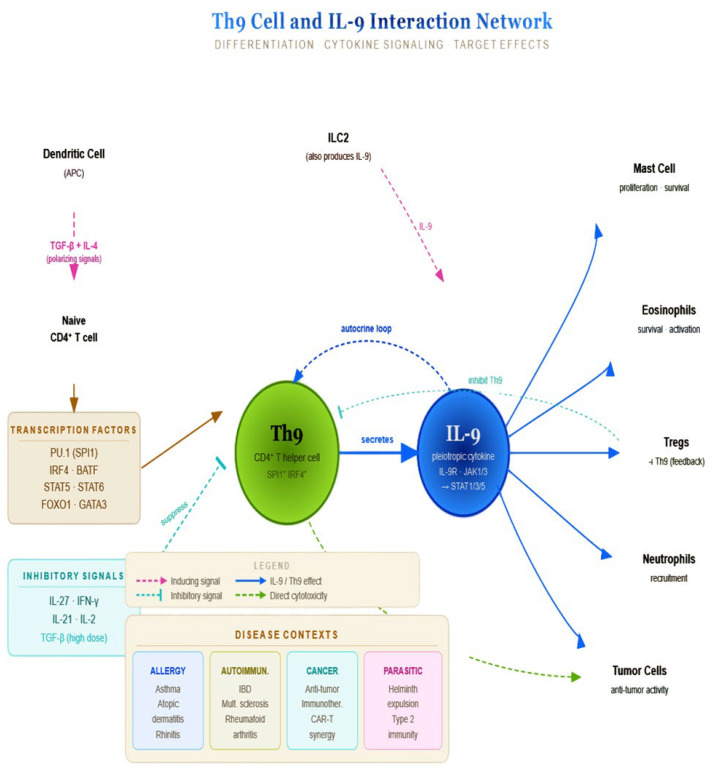
Chart depicting IL-9–mast cell crosstalk. Th9 differentiation from naïve CD4^+^ T cells is driven by TGF-β and IL-4 and inhibited by IL-27, IFN-γ, IL-21, IL-2, and high-dose TGF-β. Th9-derived IL-9 signals via IL-9R–JAK–STAT pathways to regulate mast cells, eosinophils, Tregs, neutrophils, and tumor cells, with roles across allergy, autoimmunity, cancer immunity, and helminth responses. APC—antigen-presenting cell; ILC2—group 2 innate lymphoid cell; TGF-β—transforming growth factor-β; IL—interleukin; IFN-γ—interferon-γ; STAT—signal transducer and activator of transcription; JAK—Janus kinase; Treg—regulatory T-cell; IBD—inflammatory bowel disease; CAR-T—chimeric antigen receptor T-cell.

IL-9 and mast cells form a powerful bidirectional amplification loop, particularly central to type-2 immunity, allergic disease, and parasitic infection responses, being one of the most potent mast cell growth and activation factors known regarding proliferation and survival (via IL-9R/JAK1/STAT1/3/5 signaling), synergizing with SCF (stem cell factor), differentiating (the maturation of mast cell progenitors and increasing connective tissue and mucosal mast cell populations), priming for activation (upregulating FcεRI expression, lowering the threshold for IgE-mediated degranulation), and enhancing mediator release (histamine, tryptase, leukotrienes, and prostaglandins upon allergen challenge) and cytokine induction (IL-6, TNF-α, and additional IL-9). Mast cells actively shape IL-9-producing cell populations in a number of ways: mast cell-derived IL-2 supports the differentiation of Th9 cells (the primary IL-9 source) and ILC2s; TNF-α and SCF create a microenvironment favorable for IL-9 secretion by T cells, and ILC2s can directly produce IL-9 themselves upon IgE-mediated or SCF-driven activation, acting as the source and target [8]. Figure 7 summarizes the Th9 cell–IL-9 interaction network.

Phenotype- or stage-specific interpretation

In Table 2, we provide a structured table distinguishing protective vs. pathogenic roles of IL-9 across disease phases or inflammatory endotypes.

Moreover, in Table 3, we have compiled a comparative table summarizing the IL-9 expression or function across airway compartments.

Therapeutic target

An IL-9 blockade might have value in specific subgroups, such as mast-cell-dominant CRS endotypes, steroid-resistant allergic disease, and diseases in which mast cells drive pathology. There is no strong clinical pipeline pursuing IL-9 therapies for AR/CRS at present. The main IL-9-targeting therapy that has been investigated clinically is enokizumab, tested in asthma. The results showed modest biologic activity with no consistent improvement in clinical outcomes, which led to discontinuation of development, suggesting that IL-9 is not a dominant driver cytokine compared with IL-4, IL-5, or IL-13 [56]. Expanding beyond enokizumab, the IL-9 therapeutic landscape includes receptor-level targeting and indirect pathway inhibition via JAK inhibitors. While current evidence suggests that an IL-9 blockade alone may have limited efficacy compared with established type-2 biologics, its role in specific endotypes and combination strategies remains promising. Future approaches integrating genetic stratification (e.g., IL9 eQTL profiles) with targeted therapies may enable the more precise modulation of this pathway (Table 4).

**Limitations**

There are various limitations of the present scoping review, the most important of which is the inclusion of only free, full-text, open-access articles that could have eluded publications that are not updated to the current publishing landscape. The access only to open articles or in databases through free institutional access granted by authors offered a partial perspective of the research in the field, depriving the authors from possible information that would have enhanced the power and significance of the material, making an argument for free access to medical news (Table 5).

## 9. Conclusions

Future research on the IL-9 axis must transition from descriptive association studies to mechanistically grounded, multi-dimensional investigations. By integrating genomics, single-cell biology, and longitudinal clinical data, it will be possible to define IL-9-specific endotypes, identify predictive biomarkers, and optimize targeted therapeutic strategies. This shift is essential to fully elucidate the role of IL-9 as a context-dependent regulator of airway inflammation and remodeling.

## Figures and Tables

**Table 1:** Summary of gaps and current status.

Context	IL-9/Th9 Usefulness
Mast cell-rich T2 AR [8]	Supportive marker
T2-high CRSwNP [32]	Adjunctive marker
Neutrophilic CRS [29]	Poor discriminator
Clinical stratification [28]	Not superior to IL-5/IL-13/eosinophils

**Table 2:** IL-9 roles.

Disease Phase/ Endotype	Main Cellular Sources of IL-9	Dominant Target Cells	IL-9 Biological Effects	Functional Outcome	Interpretation
Early allergen sensitization (type-2 initiation) [6]	Th9 cells, ILC2s, mast cells	Mast cells, dendritic cells	Mast cell expansion and survival; enhancement of antigen presentation	Amplifies early allergic sensitization	Pathogenic
Acute type-2 allergic inflammation [8]	Th9, ILC2s	Mast cells, eosinophils, epithelial cells	Increases mast cell degranulation, mucus production, epithelial cytokines	Drives airway hyperreactivity and mucus secretion	Pathogenic
Mast cell-dominant endotype [33]	Th9, mucosal mast cells	Mast cells, basophils	Positive-feedback loop promoting mast cell accumulation and IgE-mediated activation	Sustains local allergic inflammation	Pathogenic biomarker/amplifier
Eosinophilic CRSwNP/asthma [10]	Th9, ILC2s	Eosinophils, epithelial cells	Promotes chemokine production (CCL11, CCL24 indirectly), mucus hypersecretion	Supports type-2 tissue inflammation and polyp growth	Pathogenic
Barrier repair/epithelial recovery phase [29]	Regulatory T cells, Th9-like cells	Epithelial cells, innate lymphoid cells	Enhances epithelial regeneration and mucosal barrier repair	Tissue restoration after inflammation	Protective/reparative
Helminth infection immunity [1]	Th9, ILC2s	Mast cells, goblet cells	Mucus production, parasite expulsion, mast cell activation	Efficient parasite clearance	Protective host defense
Chronic inflammation remodeling phase [28]	Persistent Th9, mast cells	Fibroblasts, epithelial cells	Sustained cytokine production and mucus gene expression	Contributes to airway remodeling and chronicity	Pathogenic (chronic disease driver)
Regulatory immune phase (context-dependent) [2]	Treg-derived IL-9, Th9	Regulatory immune networks	Supports mast cells that can promote tolerance in some contexts	Immune modulation	Potentially protective (context-dependent)

**Table 3:** Upper–lower airway axis and Th9–IL-9 interplay.

Feature	Upper Airways (Allergic Rhinitis/CRSwNP) [8,10,19,29,32]	Lower Airways (Asthma) [2,6,11,12]	Shared Mechanism [1,3,29]
Main tissues involved	Nasal mucosa, paranasal sinuses, nasal polyps	Bronchial epithelium, airway smooth muscle	Continuous respiratory epithelium
Major IL-9-producing cells	Th9 cells, ILC2s, mast cells, mucosal T cells	Th9 cells, ILC2s, mast cells, Th2 cells	Type-2 immune polarization
Key IL-9 target cells	Mast cells, epithelial cells, eosinophils	Mast cells, airway smooth muscle, epithelial cells	Mast cell amplification
Dominant inflammatory pattern	Eosinophilic or mast cell-dominant inflammation	Eosinophilic type-2 asthma	Type-2 cytokine network
Functional IL-9 effects	Mast cell accumulation in nasal mucosa, mucus secretion, epithelial cytokine release	Mast cell expansion, airway hyperresponsiveness, mucus hypersecretion	Amplification of allergic inflammation
Clinical manifestations	Nasal obstruction, rhinorrhea, polyp growth	Bronchoconstriction, wheeze, airway hyperreactivity	Comorbid AR–asthma phenotype
Role in disease persistence	Supports chronic sinonasal inflammation	Contributes to airway remodeling	Systemic Th2/Th9 signaling
Potential biomarker role	Marker of mast cell-rich CRSwNP endotype	Marker of severe type-2 asthma	Shared biomarker across united airway disease

**Table 4:** Summary of therapeutic targets.

Therapy/Target	Agent	Phase	Population	Primary Endpoint(s)	Secondary Endpoint(s)	Key Outcomes
Anti-IL-9 (ligand blockade)	MEDI-528 (enokizumab)	Phase IIb RCT	Moderate–severe uncontrolled asthma (n ≈ 329)	Change in ACQ-6 score at week 13	Exacerbation rate, FEV_1_, AQLQ, safety	No significant improvement vs. placebo in ACQ-6, exacerbations, or FEV_1_ [56]
	MEDI-528	Phase IIa	Mild–moderate asthma	Symptom scores	Exacerbations, lung function	Modest trend toward reduced exacerbations; not statistically robust [56]
Anti-IL-9 (preclinical → early clinical translation)	Various anti-IL-9 mAbs	Preclinical → early-phase	Asthma models/early human studies	Airway hyperresponsiveness	Inflammation, IgE, mast cell activity	Strong efficacy in murine models; inconsistent translation to humans [57]
IL-9 receptor (IL-9R) blockade	Anti-IL-9R mAbs (experimental)	Preclinical/early development	Allergic airway inflammation models	Inhibition of IL-9 signaling	Mast cell survival, ILC2 activation	Promising mechanistic suppression; no large human trials yet reported [3]
JAK inhibition (indirect IL-9 pathway suppression)	Tofacitinib/baricitinib/upadacitinib	Phases II–III (other indications; exploratory in airway disease)	Inflammatory diseases incl. asthma subsets	Disease-specific composite endpoints	Cytokine signaling (IL-4/5/9/13), biomarkers	Broad cytokine suppression; potential to inhibit IL-9 signaling via JAK–STAT pathway (indirect evidence) [58]
Combination/network targeting (conceptual)	IL-4Rα blockade (e.g., dupilumab)	Phase III (approved indications)	Asthma, CRSwNP	Exacerbation reduction, lung function	Biomarkers (IgE, FeNO)	Indirect suppression of IL-9-driven networks via upstream Th2 inhibition [59]

**Table 5:** Summary of limitations.

Question	Current Status
Is IL-9 elevated in CRSwNP? [29,30,32]	Yes—mRNA and protein
Do neutrophils produce IL-9 in polyps? [27,31]	Yes
Is there a direct IL-9 → neutrophil recruitment mechanism shown in CRS? [8,13,33]	No direct evidence yet
Can IL-9 → mast cell → CXCL8 → neutrophil be inferred? [1,29,34]	Plausible from component studies
Is Th9/IL-9 endotyped separately in CRSwNP cohorts? [2,4,5,14,20]	Not yet systematically

## Data Availability

The data are available upon reasonable request from the corresponding authors.

## List of Abbreviations

AR	Allergic rhinitis
ASC	Apoptosis-associated Speck-like protein containing a CARD
AUC	Area under the curve
BAFF	B-cell-activating factor
BATF	Basic Leucine Zipper ATF-Like Transcription Factor
CCL	Chemokine (C-C motif) Ligand
CCR	C-C Chemokine Receptor
COPD	Chronic obstructive pulmonary disease
CNS	Conserved noncoding sequence
CRS	Chronic rhinosinusitis
CRSwNP	Chronic rhinosinusitis with nasal polyps
CRSsNP	Chronic rhinosinusitis without nasal polyps
CXCL	Chemokine (C-X-C motif) Ligand
CXCR	C-X-C Chemokine Receptor
Der p	*Dermatophagoides pteronyssinus*
ELISA	Enzyme-linked immunosorbent assay
EOS	Eosinophil
FcεRI	High-Affinity IgE Receptor
GC	Germinal center
GATA3	GATA Binding Protein 3
GM-CSF	Granulocyte–Macrophage Colony-Stimulating Factor
HDM	House dust mite
HIF-1α	Hypoxia-Inducible Factor 1 Alpha
HOTAIRM1	HOXA Transcript Antisense RNA, Myeloid-Specific 1
hCLCA1	Human Calcium-Activated Chloride Channel Regulator 1
IBD	Inflammatory bowel disease
IgE	Immunoglobulin E
IL	Interleukin
IL-1β	Interleukin-1 Beta
IL-2	Interleukin-2
IL-4	Interleukin-4
IL-5	Interleukin-5
IL-7	Interleukin-7
IL-9	Interleukin-9
IL-9R	Interleukin-9 Receptor
IL-13	Interleukin-13
IL-15	Interleukin-15
IL-17A	Interleukin-17A
IL-18	Interleukin-18
IL-21	Interleukin-21
IL-22	Interleukin-22
IL-27	Interleukin-27
IL-33	Interleukin-33
ILC	Innate lymphoid cell
ILC2	Type-2 innate lymphoid cell
ILC9	IL-9-producing innate lymphoid cell
IRF4	Interferon-Regulatory Factor 4
JAK	Janus Kinase
LC-MS/MS	Liquid chromatography–tandem mass spectrometry
LAR	Local allergic rhinitis
LPS	Lipopolysaccharide
LM	Lund–Mackay score
MAIT	Mucosal-associated invariant T-cell
MC	Mast cell
mRNA	Messenger Ribonucleic Acid
mTOR	Mechanistic Target of Rapamycin
NAC	Nasal allergen challenge
NALT	Nasal-associated lymphoid tissue
NF-κB	Nuclear Factor Kappa-B
NK	Natural killer cell
NLRP3	NOD-Like Receptor Family Pyrin Domain-Containing 3
NOD	Nucleotide-Binding Oligomerization Domain
OVA	Ovalbumin
OXPHOS	Oxidative phosphorylation
PBMC	Peripheral blood mononuclear cell
PHA	Phytohemagglutinin
PNIF	Peak nasal inspiratory flow
PRISMA	Preferred Reporting Items for Systematic Reviews and Meta-Analyses
PU.1	ETS Family Transcription Factor PU.1
qPCR	Quantitative polymerase chain reaction
RA	Rheumatoid arthritis
RAST	Radioallergosorbent test
RQLQ	Rhinoconjunctivitis Quality of Life Questionnaire
ROC	Receiver operating characteristic
SCF	Stem cell factor
SDHA	Succinate Dehydrogenase Complex Flavoprotein Subunit A
SEB	Staphylococcal Enterotoxin B
SGK1	Serum- and Glucocorticoid-Induced Protein Kinase 1
SLIT	Sublingual immunotherapy
SNOT-22	Sino-Nasal Outcome Test-22
STAT	Signal Transducer and Activator of Transcription
SUCNR1	Succinate Receptor 1
TCR	T-Cell Receptor
TDI	Toluene Diisocyanate
Tfh	T follicular helper cell
Tfr	T follicular regulatory cell
TGF-β	Transforming Growth Factor Beta
Th	T helper cell
Th0	Naïve T helper cell
Th1	T helper 1 cell
Th2	T helper 2 cell
Th9	T helper 9 cell
Th17	T helper 17 cell
TL1A	TNF-Like Ligand 1A
TNBC	Triple-negative breast cancer
TNF-α	Tumor Necrosis Factor Alpha
TSLP	Thymic Stromal Lymphopoietin
TSLPR	Thymic Stromal Lymphopoietin Receptor
UC	Ulcerative colitis
VAS	Visual analog scale
WGE	Wild grape extract
γc	Common Gamma Chain
